# A murine model of early *Pseudomonas aeruginosa* lung disease with transition to chronic infection

**DOI:** 10.1038/srep35838

**Published:** 2016-11-02

**Authors:** H. K. Bayes, N. Ritchie, S. Irvine, T. J. Evans

**Affiliations:** 1 Institute of Infection, Immunity and Inflammation, University of Glasgow, UK

## Abstract

*Pseudomonas aeruginosa* (PA) remains an important pathogen in patients with cystic fibrosis (CF) lung disease as well as non-CF bronchiectasis and chronic obstructive airways disease. Initial infections are cleared but chronic infection with mucoid strains ensues in the majority of CF patients and specific interventions to prevent this critical infection transition are lacking. The PA bead model has been widely used to study pulmonary *P.aeruginosa* infection but has limitations in animal husbandry and in accurately mimicking human disease. We have developed an adapted agar bead murine model using a clinical mucoid strain that demonstrates the key features of transition from transitory to chronic airways infection. Infected animals show very limited acute morbidity and mortality, but undergo infection-related weight loss and neutrophilic inflammation, development of anti-pseudomonal antibodies, variable bacterial clearance, endobronchial infection and microbial adaptation with PA small colony variants. We anticipate this model will allow research into the host and microbial factors governing this critical period in *Pseudomonas aeruginosa* pulmonary pathogenesis when transition to chronicity is occurring.

*Pseudomonas aeruginosa* (PA) remains an important pathogen in patients with cystic fibrosis (CF), as well as causing disease in non-CF bronchiectasis^1^ and chronic obstructive airways disease (COPD)^2^,^3^. Persistent PA infection also commonly complicates lung transplantation with associated poorer long-term outcome^4^,^5^. Persistent pulmonary PA infection has been most widely studied in CF. Initially infections are cleared by host responses and anti-microbial therapy, but the majority of patients transition to chronic infection with biofilm-forming mucoid strains of PA that cannot be eradicated^6^. Persistent pulmonary infection is associated with a neutrophil-dominated host response, progressive respiratory function decline and reduced patient survival in CF^7^. Therefore a ‘window of opportunity’ exists to prevent transition to chronic infection. Current strategies can delay infection but interventions that truly prevent establishment of chronic PA infection, including anti-pseudomonal vaccines, have remained elusive^8^.

Lack of representative models of human lung disease due to PA infection has been a significant bar to research. Murine models of CF lung disease, with mutated CF transmembrane receptors (CFTR), fail to develop spontaneous and chronic PA infection^9^,^10^. Porcine and ferret models with mutant CFTR hold promise^11^,^12^ but remain in their infancy and lack of reagents, particularly in immunobiology, limit use. Administration of free-living *Pseudomonas* to the murine lung results in either rapid bacterial clearance or acute overwhelming sepsis^9^. To mimic persistent infection, PA must be inoculated within an immobilizing agent. Cash *et al*.^13^ originally described a model utilizing PA-laden agar beads in rats. Embedding PA within beads allows bacterial retention, protected from physical and immune elimination, so mimicking the bacterial biofilm seen within the lung in chronic infection.

The agar bead model is technically demanding, including requirement for surgical transtracheal instillation of bead suspension^14^. High procedure-related mortality rates exist with up to 45% intra-operative mortality reported^14^, although many studies fail to report such animal losses. The majority of studies utilize injectable anaesthesic agents.

The agar bead model has predominantly been advocated as a mimic of established chronic *Pseudomonas aeruginosa* infection^9^,^15^. However the majority of studies utilize early time-points with a high level of acute inflammation and infection. Recently a model of chronic infection with established pulmonary epithelial degeneration, collagen deposition and elastin degradation has been described and more accurately represents established lung disease^15^. At the time of writing, modeling the ‘window of opportunity’ where transition to chronic *Pseudomonas aeruginosa* infection is occurring has not been described. Following refinement of the surgical technique we describe our observations using a clinically relevant mucoid PA, strain NH57388A, to develop a model that is representative of the critical transition period where chronic infection is becoming established in the lungs of patients.

## Materials and Methods

### Ethical Statement

The animal studies were approved by the granting of a project license from the UK Home Office, a ministerial UK government department that oversees all experimental work with animals in the UK. The project license number is 60/4361. All housing, maintenance and experimentation on animals used in these studies is thus in accordance with and fully compliant with the UK Government “Animals (Scientific Procedures) Act 1986” as revised (2013) to incorporate European Directive 2010/63/EU on the protection of animals used for scientific purposes. This work was also reviewed and approved by the University of Glasgow Animal Welfare and Ethical Review Board, under the same license number.

### Animals

C57BL-6 mice, aged 12–16 weeks old, were used (obtained from Harlan Laboratories and maintained at the University of Glasgow, UK).

### PA-laden agar beads

Tryptic soya agar beads laden with PA were produced as described previously^14^. The mucoid PA strain NH57388A, derived from the sputum of a patient with CF, was utilized (kindly provided by N. Hoffmann, University of Copenhagen). NH57388A-laden agar beads were prepared the day before inoculation, stored overnight at 4 °C, suspended in sterile PBS to deliver approximately 1 × 10^6^ CFU in 50 μl per mouse. The inoculation dose was chosen following initial experiments demonstrating high acute mortality with higher inoculation doses. Different bead preparations were used for each experiment. Following PA-laden beads inoculation, the administrated inoculum was confirmed by quantitative bacteriology. Sterile agar beads were used for several experiments and confirmed as sterile before and after each use.

The model was developed and phenotyped in 82 mice; typically 10 mice per group used in a given experiment with 34 mice treated with sterile beads and 48 treated with NH57388A-laden agar beads. Ongoing work examining immune responses to PA infection (total 300 animals treated with both sterile and PA-laden agar beads) provided greater experience of true operative mortality.

### Trans-tracheal instillation of beads

**Figure 1:**
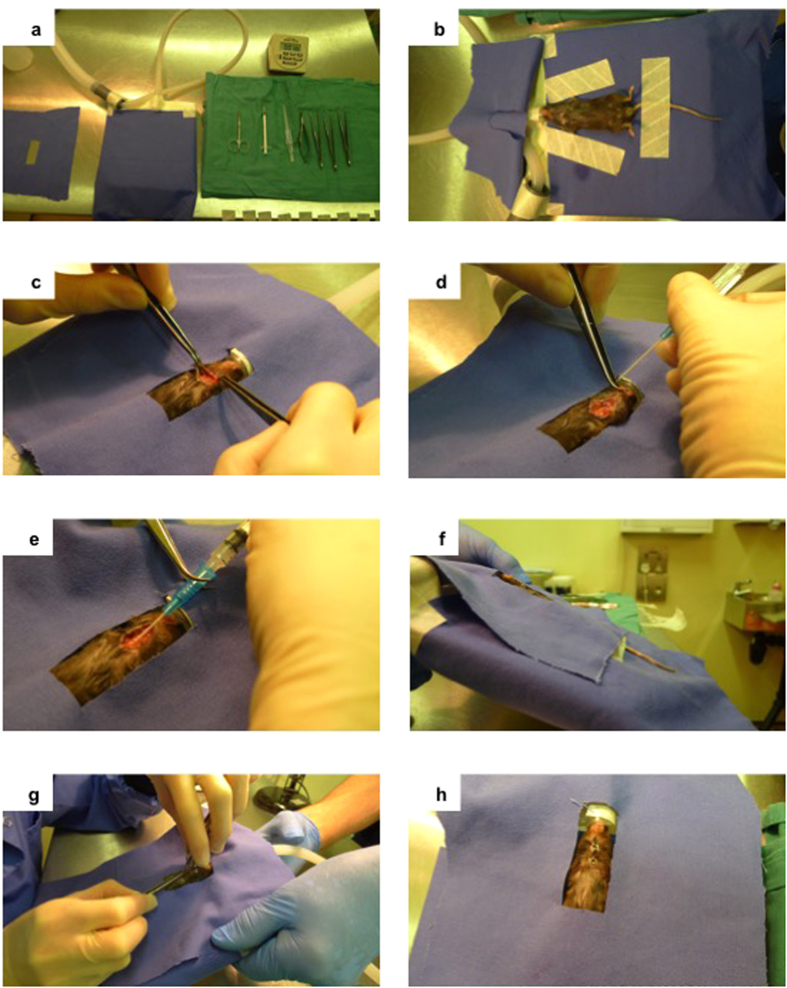
Technique for intra-tracheal instillation of agar beads using inhaled anaesthesia. (**a**) Sterile surgical equipment set-up required for instillation. (**b**) Following anesthesia administered via isofluorane inhalation in an anesthesia box, animals were transferred to the surgical board and maintained under anaesthesia via nose cone. (**c**) Midline neck incision and careful division of soft tissues to expose trachea. (**d**) Insertion of 22G intravenous cannula into trachea, while extending neck using traction on lower jaw. (**e**) Injection of 50 μl of beads/PBS via cannula. (**f**) Elevation of anaesthetized animal on surgical board head-up to 45-degrees for a timed 1-minute in order to aid full inspiration of beads. (**g**) Closure of incision with surgical staples. (**h**) Completed closure of incision.

The technique for trans-tracheal instillation of beads is detailed in Fig. 1. Mice were anaesthetized with inhaled isoflurane delivered initially in an anaesthetic box until unconscious, transferred to a surgical board and maintained under anesthesia with isoflurane via nose cone (Fig. 1b). The ventral cervical region was sterilized and 1-cm midline skin incision made just cranial to the thoracic inlet. The trachea was visualized by blunt dissection (Fig. 1c). Traction on the lower jaw was used to extend and straighten the trachea and a 22G intravenous catheter (BD Biosciences) used to cannulate the trachea (Fig. 1d). A 1-ml syringe, pre-filled with beads suspended in PBS, was attached to the cannuale (Fig. 1e). The cannulae and syringe were left *in-situ* for 5-seconds following depression of the plunger, followed by slow withdrawal. The animal was elevated head up to 45-degrees (Fig. 1f). A rapid rise in respiratory rate was frequently observed at this point suggesting accurate intrapulmonary bead instillation. If evidence of respiratory distress (rapid or labored respiration/gasping) occurred during the procedure anesthesia was rapidly reversed and oxygen alone administered via nose cone. The incision was closed with surgical staples (Fig. 1g,h). Buprenorphine analgesia (Vetergesic; Alstoe Animal Health) was administered subcutaneously post-operatively.

### Post-operative care

Animals were monitored post-operatively in a heat box until ambulant and clinically normal. Mice were transferred to a clean box with food and water ad libitum and monitored at 1-hour, 4-hours and 8-hours post-operatively using a clinical scoring system for disease severity. Animals were weighed and scored clinically daily. Animals reaching a moribund end-point or with weight loss of greater than 20% of baseline weight were terminated prior to the pre-defined experiment end-point. Experiments were terminated at 2-weeks post-inoculation via carbon dioxide asphyxiation.

Blood, bronchoavlaeolar lavage (BAL), pulmonary lymph nodes and lung tissue were harvested from each animal under aseptic conditions. 1-ml of sterile phosphate buffered saline (PBS) infused and aspirated into the airways three times for each BAL sample. Thoracic lymph node cells were obtained from the bilateral mediastinal lymph nodes and single tracheobronchial lymph node^16^. The lung was divided based on given lobes for different outcome measures: right apical lobe for myeloperoxidase (MPO) assay, right diaphragmatic lobe for microbiology, and left lung inflation fixed for histology in zinc fixative (BD Pharmingen).

### Bacteriology

Lung tissue was mechanically homogenized in 1-ml PBS. Blood, BAL and lung homogenates were plated for quantitative bacteriology on Luria-Bertani (LB) agar (Invitrogen) and examined after 24 and 48 hours incubation. Colonies were confirmed to be PA by appearance, Gram stain (BD Biosciences) and oxidase testing (Sigma-Aldrich). Smaller atypical colonies, known as PA small-colony variants (SCV)^17^,^18^, were confirmed as *Pseudomonas* species via VITEK2^®^ identification. Chronic infection was defined as recovery of mucoid PA from lung cultures at 2-weeks post-inoculation.

For transmission electron microscopy (TEM), bacterial strains were grown on a coverslip in a petri-dish with LB broth at 37 °C for 36-hours. These were then washed in PBS, processed and stained as previously described^19^.

### Measurement of immunological parameters

#### Neutrophil quantification

Blood and BAL samples underwent red blood cell (RBC) lysis (Red cell lysis buffer; Sigma-Aldrich) prior to staining with Gr-1 (Ly6G; RB6-8C5; BioLegend) for flow cytometry (FACS Aria; BD Biosciences). MPO activity was quantified using a kit following the manufactuer’s instructions (Sigma-Aldrich).

#### Lymph node B cell quantification

Thoracic lymph nodes, passed through 80 μm nitrex mesh, underwent RBC lysis, prior to staining with CD19 (6D5; BioLegend) and B220 (RA-6B2; BioLegend) for flow cytometry.

#### Lung histology

Lungs were embedded in paraffin, sectioned at 5 μm and stained with haematoxylin and eosin. Histology was scored for peribronchial and alveolar involvement using a scoring system adapted from Dubin *et al*.^20^ (Table 1). Lung section scoring was performed at x10 magnification with an overall score given for whole lung sections and verified by independent blinded investigators.

#### PA-specific immunoglobulin quantification

At termination (2-weeks post-infection), blood was aspirated from the inferior vena cava, followed by centrifugation to obtain serum for immunoglobulin (Ig) quantification. ELISA-based detection of PA-specific IgM and IgG was utilized^21^ with binding bacterial protein derived from the NH57388A strain. Bound antibody was quantified by detection of biotinylated goat anti-mouse IgM (mu chain specific; VectorLabs) or anti-mouse IgG (Fc specifc; Sigma-Aldrich).

### Statistical analysis

Results are presented as medians or, for technical repeats, mean and standard error of mean (SEM). Non-parametric Mann-Whitney or Kruskal-Wallis tests were used. Animal weight were compared repeated-measures ANOVA. Statistical analysis was undertaken using Prism Version 6.0 (GraphPad Software).

## Results

### Operative outcome

Prior to the use of trans-tracheal instillation of beads in our model, we undertook preliminary experiments to exploring alternative means of delivering the agar beads, namely cannulation of the trachea via the oral cavity and intranasal instillation, with the aim of effective bead delivery but reduced incision-related inflammation in the model. Experiments on mice cadavers with attempted cannulation of the trachea via the mouth demonstrated high levels of insertion outside of the tracheal, particularly gastric cannulation. Secondly, bead inoculation was attempted via intranasal inhalation in animals under an inhaled isoflurane anesthetic, with inoculated animals culled 1-hour later and lung sections stained and screened for presence of agar beads. This demonstrated very infrequent, small agar beads within the airways, suggesting either lodging of the beads within the upper airways or swallowing of beads. In contrast, frequent and larger beads that were identified in lung sections of mice administered beads via the trans-tracheal route. Thus the trans-tracheal route of instillation was subsequently utilized in our model.

The adapted intratracheal method (Fig. 1) demonstrated accurate delivery and retention of PA, 100% of lungs (N = 8) infected at 24-hours post-surgery. Animals recovered rapidly post-procedure; ambulant within 10-minutes and clinically normal within 30-minutes. Overall extremely low intra-operative or early peri-operative (<1 hour post-procedure) mortality rates resulted from the adapted transtracheal method: 4 fatalities in 300 procedures (operative mortality rate 1.3%). These deaths occurred during or shortly following completion of surgery.

### Clinical effect of infection

**Figure 2:**
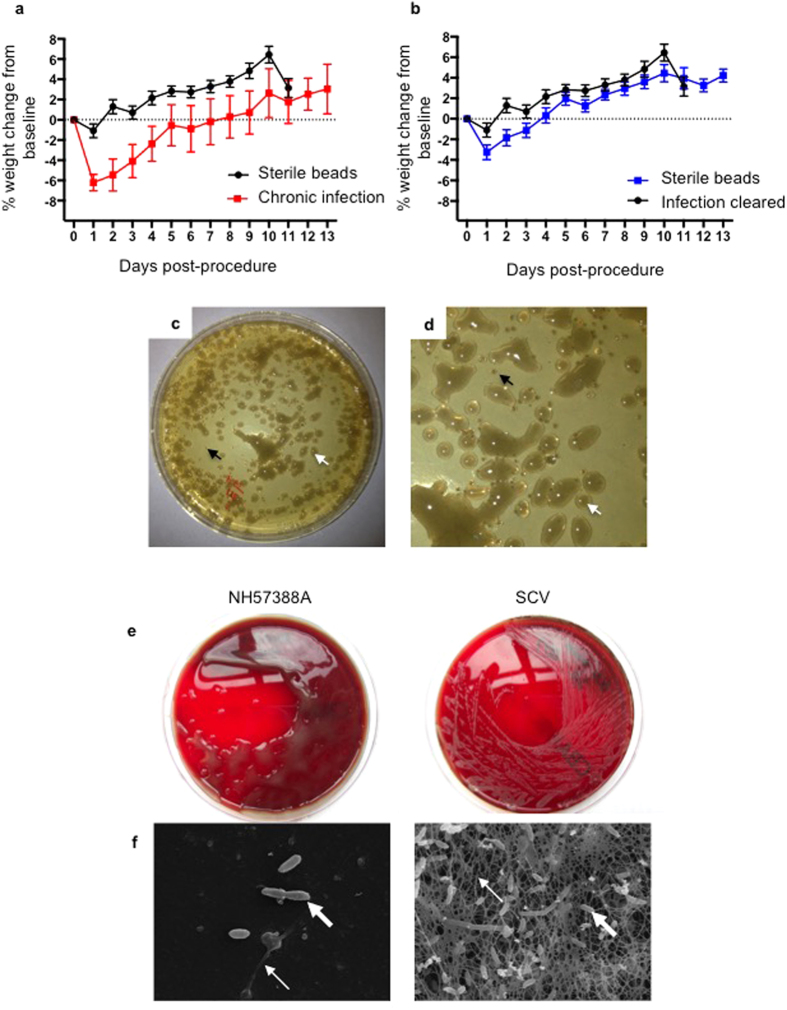
Weight loss and bacterial adaptation in mice infected with NH57388A-laden agar beads. (**a**,**b**) Body weight measured daily in C57BL/6 mice treated with sterile or *Pseudomonas aeruginosa*-laden agar beads. Animals who were infected with NH57388A-laden beads and, at 2-weeks post-inoculation, remain infected are denoted as ‘Chronic infection’ (**a**), where as those who do not have pulmonary infection are denoted ‘Infection cleared’ (**b**). Percentage weight change from baseline for pooled experiments with animals treated with NH57388A-laden beads (N = 35) compared with controls treated with sterile beads (N = 30 mice). Each point represents mean of group and SEM. (**a**) Difference between groups significant at p < 0.0001 via repeated measures ANOVA. (**b**) Difference between groups significant at p = 0.0204 via repeated measures ANOVA. (**c**,**d**) Mucoid (white arrow) and small colony variants (black arrow) identified in lung homogenate of NH57388A-laden bead treated animal 2-weeks following inoculation. (**e**) Subcultures of mucoid NH75388A and small colony variant (SCV) phenotypes isolated from the lungs of animals treated with NH57388A-laden beads (grown on blood agar). (**f**) Transmission electron microscopy of biofilms of mucoid NH57388A and its small colony-variant. Mucoid NH57388A demonstrated low bacterial density with limited extracellular matrix. The related SCV produced a biofilm with a high density of highly adherent bacteria embedded in a dense network of extracellular material. Bacteria indicated by think white arrow and extracellular matrix by thin white arrow.

The majority of mice remained clinically normal throughout. Post-procedure weight loss was significantly greater and sustained in animals developing chronic NH57388A pulmonary infection compared with sterile controls (Fig. 2a; p < 0.0001, via repeated measures ANOVA). Animals treated with PA-laden beads who cleared infection had early weight loss but returned to weights similar to sterile bead treated mice within 5-days (Fig. 2b).

### Establishment of persistent infection

High acute mortality (>1-hour post-procedure but before the 2-week defined experimental endpoint) occurs in pseudomonas pneumonia^22^ but is not a feature of the transition to chronic pulmonary PA infection in the human population. Correspondingly limited acute mortality occurred in animals treated with NH57388A-laden beads (3 mice, 6% of total NH58773A-inoculated mice); culled due to weight loss >20% of baseline and demonstrated high pulmonary bacterial burden (mean 3.6 × 10^9^ CFU in lung homogenates, SD: 9.4 × 10^9^ CFU). No acute mortality occurred in sterile bead treated mice.

Overall, NH57388A-laden bead inoculation resulted in mean chronic PA infection rates at 2-weeks of 37.3% (SD: 18.3%; range: 11.1–60%). Median pulmonary PA burden at 2-weeks was 1355 CFU (total of BAL and right diaphragmatic lobe homogenate; IQR: 182–2898). No *P. aeruginosa* was isolated from animals treated with sterile agar beads. No bacteria were isolated from terminal blood cultures of any animal.

### Bacterial adaptation

Lung homogenates and BAL samples obtained from animals chronically infected with NH57388A demonstrated two distinct *Pseudomonas aeruginosa* colony morphologies: large mucoid colonies identical in morphology to the inoculating strain and smaller atypical small-colony variants (SCVs) (Fig. 2c–f). Small colony variants are very well described as part of the phenotypic adaptations to the CF lung^23^. Mucoid colonies were evident after 24-hours, whereas SCVs were only visible after 48-hours of incubation. TEM analysis of the SCV and its parent strain NH57388A demonstrated marked differences in biofilm formation (Fig. 2f). The biofilms of mucoid NH57388A demonstrated low bacterial density within the limited extracellular matrix. In contrast, the related SCV produced a biofilm with a high density of highly adherent bacteria embedded in a dense network of extracellular material (Fig. 2f). This complex and adherent biofilm is likely a key factor in the ability of SCVs to produce lung pathology.

Overall SCVs were present in 12 of 17 animals (70.6%) with chronic NH57388A infection. To ascertain that the SCV development was not due to embedding in beads alone, NH57388A-beads were cultured intact and homogenized between 24-hours to 2-weeks after bead formation with no evidence of SCV development, repeated for 3 separately produced bead batches. In addition, culture of NH57388A from stock demonstrated only mucoid morphology even with prolonged culture. Thus *in vitro* pressures and environment within the model appear to be required for SCV development.

### Neutrophilic inflammation

**Figure 3:**
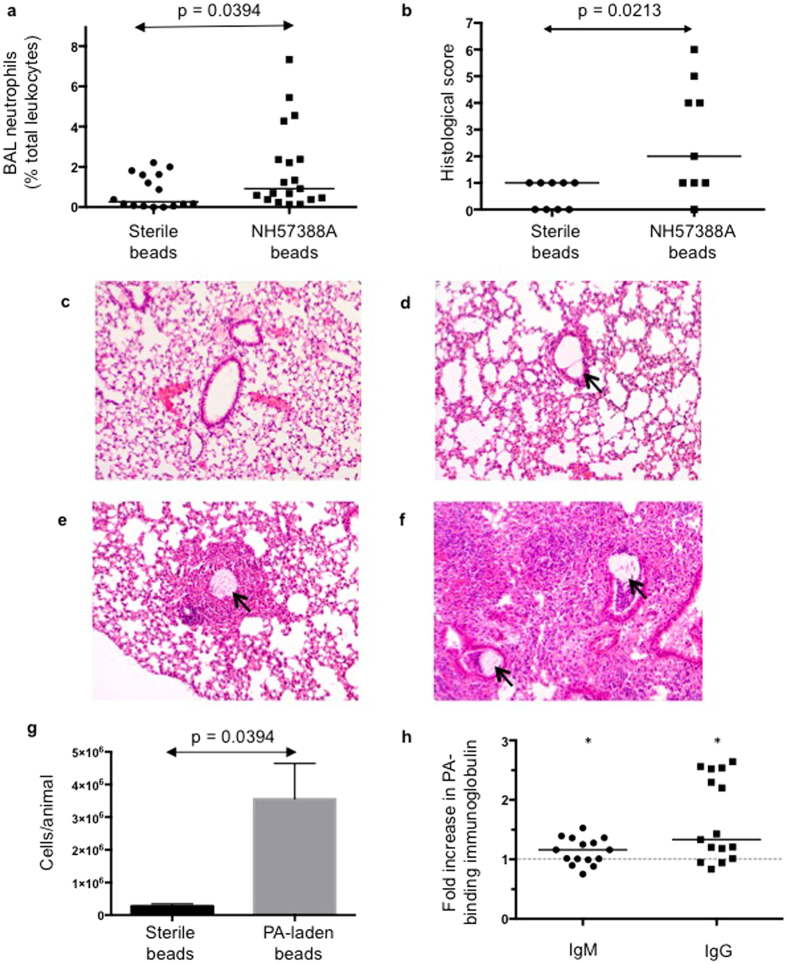
Neutrophilic inflammation, histological changes and *Pseudomonas*-binding immunoglobulin in mice two-weeks following NH57388A-laden agar bead inoculation. C57BL/6 mice received sterile or NH57388A-laden agar beads. (**a**) Two-weeks post-instillation, bronchoalveolar lavage (BAL) leukocytes were strained for Gr-1 followed by flow cytometry. BAL neutrophils (percent of total leukocytes) in animals treated with sterile beads (N = 16) were compared mice inoculated with NH57388A-laden agar beads (N = 19) at 2-weeks. Line indicates median. P-value related to Mann-Whitney test. (**b**) Quantitative histological scores of animals treated with sterile agar beads (N = 9) or NH57388A (NH)-laden beads (N = 9). Line denotes median score. P-value related to Mann-Whitney test. **(c–f**) H&E staining of representative lung sections (all at x10 magnification) in C57BL/6 mice with no treatment (healthy control) (**c**), 2-weeks post-inoculation with sterile agar beads (**d**), 2-weeks post-inoculation with NH57388A-laden agar beads (**e**), or with overwhelming acute infection post-inoculation with PA-laden agar beads. Black arrows highlight intra-bronchial agar beads. (**g**) Mediastinal lymph node cell counts in mice treated with sterile and PA-laden agar beads two-weeks following bead instillation. Columns shown mean + SD. P-value relates to comparison by t-test and includes results from 3 experiments (each with N = 9–10 mice/group). (**h**) Serum PA-binding immunoglobulin M (IgM) and G (IgG) levels showing fold increase in IgM and IgG levels in NH57388A treated animals compared with sterile bead treated controls two-weeks post-instillation. Line represents median. *Denotes p < 0.05 compared with theoretical median of 1.0 via Wilcoxon Signed rank test. Combined results from two separate experiments.

Neutrophil inflammation is consistently implicated in CF lung disease complicated by chronic PA infection where such neutrophilic responses fail to clear the organism but contribute to host damage^24^. Pediatric CF studies have demonstrated elevation of pulmonary inflammatory markers only in the presence of infection^25^. In keeping with these clinical observations, high levels of pulmonary neutrophilic inflammation were seen 48-hours following inoculation of NH57388A-laden agar beads (median BAL neutrophils (percent of total leukocytes) 79.9% (IQR: 57.3–90.2)) compared with sterile bead controls at the same time-point (median BAL neutrophils 1.76% (IQR: 0.84–6.84)). Importantly, pulmonary neutrophilic inflammation persisted at two-weeks post-inoculation with PA infection (Fig. 3a). Inflammation returned towards normal once infection cleared; median BAL neutrophils in chronically infected animals being 5.01% of total leukocytes compared with 0.68% in animals clearing PA prior to 2-weeks (p = 0.0010 via Mann-Whitney test). There was no significant increase in total lung tissue MPO activity in PA infected animals compared with sterile controls (data not shown). Peripheral blood neutrophils were similarly not significantly elevated at 2-weeks post-inoculation with PA-laden beads compared with sterile controls (data not shown), consistent with confined intrapulmonary sepsis and inflammation.

### Intra-pulmonary immune response

Persistent pulmonary inflammatory changes were evident 2-weeks post-inoculation with higher histological scoring in PA treated animals (Fig. 3b). A range of histological scores was seen in infected animals, likely reflecting the localized histological changes at sites of infection and random sampling of these. Minimal changes were seen in response to agar bead instillation *per se* (Fig. 3d). Infected lungs demonstrated localized peribronchial monocytic infiltrates with an agar bead frequently evident in the adjacent airway (Fig. 3e). This localized infiltrate was in contrast to the intense parenchymal inflammation seen in animals succumbing acutely with high bacterial burden (Fig. 3f).

### Systemic anti-pseudomonal immunoglobulin production

NH57388A infection resulted in macroscopic pulmonary lymph nodes enlargement at 2-weeks post-inoculation, confirmed quantitatively on cell counts (Fig. 3g). *Pseudomonas aeruginosa*-specific immunoglobulins (Ig) are produced by CF patients and levels have been found to increase progressively as chronic PA infection develops^26^,^27^. Serum PA-binding immunoglobulins significantly increased 2-weeks post-inoculation of NH57388A, with a median fold increase in PA-binding IgM of 1.162 (IQR: 0.994–1.362) and IgG of 1.334 (IQR: 1.101–2.521) compared with sera of sterile bead treated animals (p < 0.0001 for both, compared with theoretical median of 1.0 via Wilcoxon signed rank test) (Fig. 3h).

## Discussion

Developing models akin to human disease is essential to any research field but has remained problematic in cystic fibrosis pulmonary disease. The agar bead model has been advocated as reproducing the lung pathology of patients with CF with advanced and established chronic pulmonary infection^9^,^14^. However, the NH57388A-laden agar bead model described in this paper has features akin to the critical stage in CF lung disease when there is non-resolution of mucoid *Pseudomonas aeruginosa* infection and transition to chronic infection occurs. Our murine model demonstrated clinical, immunological and microbiological features mirroring that of human lung disease seen with the development of persistent PA infection: low levels of morbidity, weight loss related to infection, endobronchial neutrophilic inflammation and infection, adaptive immunity with production of pseudomonas-binding Ig, variable clearance of a clinical mucoid PA strain and microbial adaptation^12^,^17^,^24^,^26^,^28^.

Progression of lung disease in CF with irreversible changes and bacterial colonization is usually insidious. Our described model does not attempt to reflect CF lung disease from its onset as multiple additional infective, including infection with *Staph. aureus*, and inflammatory insults beginning early in life and precede PA infection. However, we demonstrate non-resolution of mucoid PA in a clinical relevant strain. Overwhelming sepsis is not a typical feature of early PA infections and thus, importantly, aggressive infection and acute mortality was limited in our described model. However, patients frequently manifest poor growth and nutritional status has increasingly been recognized as an important determinant of pulmonary health and survival^29^. CF-related pancreatic disease contributes but pulmonary PA infection and the associated inflammation also drives nutritional failure^30^. Our model demonstrated significant weight loss in relation to infection, which peaked acutely but remained sustained at least 10-days post-infection (Fig. 2a), with the potential to investigate interventions against this important cause of patient morbidity.

Neutrophilic inflammation in response to persistent *Pseudomonas aeruginosa* infection has been consistently implicated in pathogenesis of CF^24^,^25^. Indeed sustained endobronchial neutrophilic inflammation was evident (Fig. 3a). The fact that there was no increase in whole lobe MPO in our model would be consistent with inflammation predominantly confined to intra/peri-bronchial regions rather than inducing a significant parenchymal/pneumonia-like infection. The role of B and T lymphocyte immunity in anti-Pseudomonal immunity has not been well-characterized to-date. We found significant regional lymph node enlargement (Fig. 3g) and systemic anti-pseudomonal antibody (Fig. 3h) consistent with lymphocytic recruitment and adaptive immune activation. We have subsequently utilized the model to interrogate the role of inflammatory cytokine responses in protecting against chronic infection^31^ and defining novel cytokine-producing B cells recruited during infection (manuscript submitted, Bayes *et al*.). Further work may utilize such B cell responses for novel anti-pseudomonal mucosal vaccination approaches^32^.

The described model was developed in C57Bl6 mice rather than CFTR mutant animals, which makes the model of utility to non-CF related disease. Recent comparison of the agar bead model in a more chronic infection than described in our manuscript, demonstrated that persistent immune responses and airway remodeling were similar in CFTR mutant versus wild-type controls^15^. Whether the period of transition to chronicity is however adversely effected by exacerbated inflammatory responses or defective PA clearance requires further interrogation.

Others have previously utilized clinical CF strains of PA in agar bead model^33^. We utilised the clinical *P. aeruginosa* NH57388A strain which has a mutation in *mucA* resulting in alginate hyper-production^34^; relevant as alterations in *mucA* are the most frequently identified mutations in mucoid Pseudomonas isolates from CF patients^35^. NH57388A has previously been embedded in alginate in CF and BALB/c mice^34^, yet this alternate model resulted in significant acute mortality and limited evidence provided to suggest persistent infection beyond 7-days. Embedding NH57388A in agar beads prevented significant acute mortality, produced chronic infection rates of approximately 40% and pulmonary bacterial burden comparable to that achieved by others utilizing alternative PA strains^9^,^36^.

To mimic human disease, experimentally instilled *P. aeruginosa* should proliferate and adapt within the lung. The high bacterial burden in animals succumbing acutely and the development of small-colony variants confirms that PA has actively proliferated and adapted *in vivo*. Development of SCVs is particularly relevant to human CF disease^23^. Over 2-year follow-up, *P. aeruginosa* SCVs were isolated from 38% of adult CF patients infected with the organism and associated with poorer lung function and increased antibiotic usage^37^. Functionally, PA SCVs demonstrate features favoring persistent infection including increased biofilm formation^17^,^38^, adherence^17^, macrophage cytotoxity^38^, and antibiotic resistance^37^. These factors would suggested an ability of SCVs to evade host responses rather than showing increased virulence per se. We found no evidence of systemic spread of PA including in animals with intrapulmonary PA SCVs. Whether PA SCVs demonstrate increased virulence could be further examined in the model by infection with PA SCVs embedded in agar beads compared infection with the mucoid parent strain from the onset. Thus the described model opens up exciting opportunities to explore the genetic and environmental determinants of SCV development and investigate therapeutic targeting of this PA subtype.

Although *Pseudomonas aeruginosa* is commonly identified as a CF pathogen, the organism complicates and adversely effects outcome in the broader respiratory population. In non-CF bronchiectasis and chronic obstructive airways disease (COPD) persistent infection is associated with more rapid decline in lung function, anti-pseudomonal antibody formation as well as the PA isolates demonstrating features seen in CF disease with mucoid phenotype, increased antibiotic resistance and biofilm formation^1^,^2^,^3^. Persistent PA infection in allograft lungs has been consistent identified as a risk factor for chronic rejection, namely bronchiolitis obliterans syndrome^4^,^5^. Thus our described modeling transition to chronic pulmonary PA infection has utility in wider respiratory disease.

Improvements in laboratory animal welfare remain of paramount importance in disease model development. To this end we describe in detail our method for using inhaled anesthesia to allow transtracheal intubation of mice, with the advantages of reliable and easily reversible sedation if respiratory compromise develops during the procedure and rapid post-operative recovery. Despite use by previous authors, we are unaware of this technique being described in detail to-date that allows replication nor to demonstrate the potential benefits on operative mortality. Resultant intra-/perioperative mortality was extremely low (overall 1.3%) in our model, significantly lower than previously reported using injectable agents^14^. Although operative outcome is dependent on user experience and skill^14^, the low procedure-related mortality was consistent throughout our use of the model (i.e. was not related to mounting experience in the technique; data not shown). Organism virulence also effects outcome^33^ but would not be relevant to outcome described here given that we found the majority of peri-operative deaths occurred during/very close to the time of procedure. Importantly the model adaptions are in concordance with the principle of “refinement” in animal research both by improving operative outcome and by describing clear features akin to human disease.

A limitation of the model is in mimicking established CF lung disease with the associated structural changes of mucus plugging, gas trapping and bronchiectasis. Although the NH57388A-agar bead model demonstrated greater histological changes compared with sterile bead controls, this was limited to peribronchial monocytic infiltrates (Fig. 3). However arguably it is not possible to replicate in short-lived murine models a process that, in man, requires years of multiple infective and inflammatory insults to develop. Thus our approach to mimicking the transition period to chronic colonization in the CF lung appears more realistic. The stability of infection beyond 2-weeks was not examined, although others have demonstrated static bacterial burden beyond 2-weeks using alternate murine agar bead models^9^.

*Pseudomonas aeruginosa* remains an important respiratory pathogen, particularly in its ability to evade host and therapeutic strategies to establish chronic infection. The described NH57388A-agar bead model will allow interrogation of the microbiology and immune mechanisms during the critical ‘window of opportunity’ prior to established chronic *Pseudomonas* infection.

## Additional Information

**How to cite this article**: Bayes, H. K. *et al*. A murine model of early *Pseudomonas aeruginosa* lung disease with transition to chronic infection. *Sci. Rep.* **6**, 35838; doi: 10.1038/srep35838 (2016).

**Publisher’s note:** Springer Nature remains neutral with regard to jurisdictional claims in published maps and institutional affiliations.

## Figures and Tables

**Table 1:** Histological scoring system for inflammation in lungs of mice treated with sterile agar beads and beads laden with *Pseudomonas aeruginosa.*

Score	Peribronchial infiltrate	Alveolar involvement
0	None	None
1	Mild (infiltrate ≤ 4 cells thick)	Mild (patchy increased cellularity/thickening)
2	Moderate (infiltrate 5–10 cells thick)	Moderate (25–50% visualized lung with increased cellularity/thickening)
3	Severe (25–50% visualized lumens)	Severe (>50% visualized lung with increased cellularity/thickening)
4	Diffuse (>50% visualized lumens)	

Randomly selected sections blindly scored with a score applied to review of a whole lung section, scored at x10 magnification.
